# Bentonite Modified by Allylamine Polymer for Adsorption of Amido Black 10B

**DOI:** 10.3390/polym11030502

**Published:** 2019-03-15

**Authors:** Wenjuan Guo, Tingcheng Xia, Meishan Pei, Yankai Du, Luyan Wang

**Affiliations:** 1 Institute of Surface Analysis and Chemical Biology, University of Jinan, Jinan 250022, China; chm_guowj@163.com; 2 Shandong Labor Vocational and Technical College, Jinan 250022, China; xiatingcheng@163.com; 3 School of Chemistry and Chemical Engineering, Shandong Provincial Key Laboratory of Fluorine Chemistry and Chemical Materials, University of Jinan, Jinan 250022, China; 1287858357@163.com (Y.D.); chm_wangly@ujn.edu.cn (L.W.)

**Keywords:** bentonite, adsorbent, polyallyl amines, Amido black 10B

## Abstract

The main object of this work is to remove Amido black 10B using a new type of bentonite-based adsorbent with cationic groups by the modification of polyallyl amines between the interlayers of bentonite. Fourier transform infrared, X-ray diffraction, thermogravimetric analysis, and scanning electron microscopy were used to characterize the functionalized bentonite. A series of batch adsorption experiments were performed. The maximum adsorption amount was 144.08 mg g^−1^ when the pH was 2 and the contact time was 120 min. In addition, the equilibrium isotherm data were analyzed using Langmuir and Freundlich isotherm models, while only the Langmuir model could provide a high correlation. Therefore, this study provided a new functionalized bentonite as a low-cost adsorbent for dye removal from water.

## 1. Introduction

Water is an important resource. With the development of industry, water pollution is getting more serious. In the realm of water pollution, the industrial wastewater caused by the discharge of dyes is a non-negligible problem. Industrial wastewater discharged into rivers and lakes, and finally into the sea, creates a huge pollution problem. The use of dyes is unavoidable, as they can be found in many industrial fields, such as textiles, papermaking, plastics, food, rubber, and cosmetics. For example, crystal violet dye, the brightest dye, can cause human bladder cancer [1]. Rhodamine B can be harmful to the environment. In California, rhodamine B is considered a carcinogenic substance, and products with rhodamine B must have a warning label on the packaging [2]. Therefore, the concentration of dyes in wastewater must be reduced to the permissible limit for the sake of human health as well as environmental safety. Amido black 10B is one of the dyes most widely used in industry [3]. Zhang et al. investigated the adsorption of Amido Black 10B from aqueous solutions onto a Zr (IV) surface-immobilized cross-linked chitosan/bentonite composite [4]. The adsorption capacity was 418.4 mg g^−1^ at natural pH value and 298 K. Wang et al. prepared porous chitosan doped with graphene oxide as a highly effective adsorbent for methyl orange and amido black 10B [5]. The adsorption capacity of AB10B was 573.47 mg g^−1^. Zhang et al. prepared a crosslinked quaternized chitosan/bentonite composite for the removal of Amino black 10B from aqueous solutions [6]. The maximum monolayer adsorption capacity was 990.1 mg g^−1^ at 298 K and natural pH in terms of the Langmuir model. Hu et al. investigated the simultaneous removal of Cr(VI) and Amido black 10B (AB10B) from aqueous solutions using quaternized chitosan-coated bentonite [7]. The maximum adsorption capacity of the modified bentonite, according to the Langmuir isotherm model, was 847.5 mg g^−1^ for AB10B at 298 K. At present, the widely used technologies include advanced oxidation processes [8], adsorption [9,10,11] membrane filtration [12], and photocatalytic degradation [13]. Those technologies are expensive because of the specialist materials required. For example, membrane filtration technology requires an expensive membrane material that is not suitable for common use [14,15,16,17]. By comparison, adsorption is an efficient and relatively economical technology for the removal of dyes from wastewater. Among the adsorbents, chitosan and its derivatives have received considerable attention as flocculants in water treatment. Several chemical modification methods to prepare chitosan-based flocculants have been reported [18]. For use in practical applications, Li et al. prepared a chitosan magnetic composite adsorbent modified with a quaternary ammonium salt for the removal of Cr(VI) and methyl orange (MO) from water. This magnetic adsorbent after saturated adsorption could be rapidly separated from water [19]. Angelova et al. assessed the biosorption of Amido black 10B dye from aqueous solutions on magnetically modified sheaths of *Leptothrix* sp. in a batch system [20]. These methods allowed convenient control and adjustment of the structures of the obtained materials to meet the different practical requirements. Activated carbon materials are also traditional adsorbents that have been used in many fields. Whereas, with the increase in demand for commercial activated carbon materials, the price of activated carbon materials is becoming more expensive. As abundant, inexpensive, and environmentally friendly materials in nature, clays have potential value as adsorbents for the removal of dyes from wastewater. Yang et al. applied an acid-treated palygorskite as an adsorbent to remove various contaminants and it showed efficient performance in the adsorption of all three cationic dyes because of electrostatic interactions [21].

**Figure 1:**
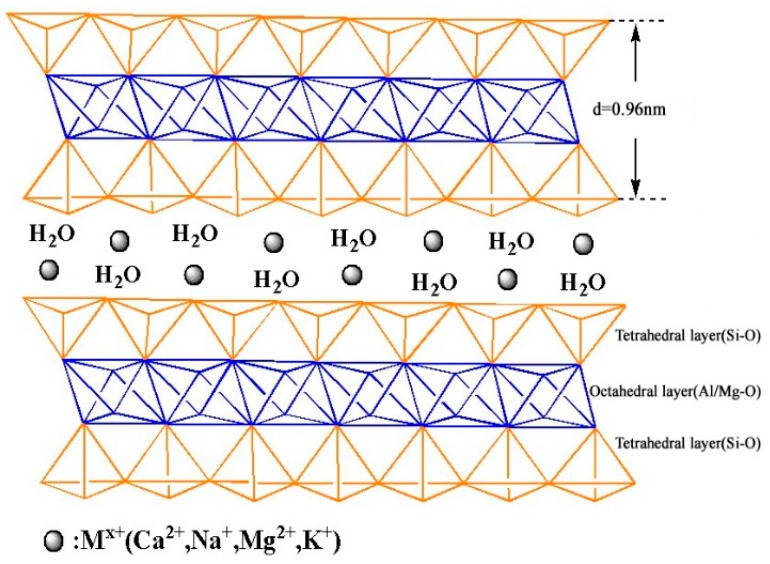
The structure of montmorillonite.

Bentonite (Bent), one of the most common clays, has been used in a variety of industrial fields as a catalyst, filler in polymer, cosmetics, and pharmaceuticals, because of its high surface area and swelling property [22]. The main clay mineral constituent of Bent is montmorillonite. Its crystal structure is shown in Figure 1: it is composed of one octahedral aluminate layer (termed as O) sandwiched between two tetrahedral silicate layers (abbreviated as T), forming a TOT type [23]. The natural Bent has limited adsorption capability, which restricts its practical applications [24]. The reason might be the poor dispersibility of Bent in water. Bent should be modified with some functional groups to improve its adsorption capability for dyes.

In this study, Bent was designed to be modified with the cationic group polyallyl amines (PAA). The as-prepared material was characterized by X-ray diffraction (XRD), Fourier transform infrared spectroscopy (FTIR), thermal gravimetric analysis (TGA), and scanning electron microscopy (SEM). The novel material was used to adsorb Amido Black 10B as a representative of the anionic dyes. Batch adsorption experiments were used to evaluate the adsorption capacity of the novel adsorbent for AB10B. Experimental data were analyzed by adsorption isotherms and adsorption kinetics.

## 2. Materials and Methods

### 2.1. Materials

Bent was supplied by Weifang Zhenxing Bentonite Co. (Weifang, China). It was purified by dispersed into water for 3 h and then centrifuged. After being dried at 80 °C in a vacuum oven, the product was obtained. Toluene, purchased from Laiyang Tieta Fine Chemical Factory (Laiyang, China), was used as the solvent. PAA, glutaraldehyde, and 3-aminopropyltriethoxysilane were obtained from Aladdin Industrial Corporation (Shanghai, China). Chemicals were analytical grade without further purification.

### 2.2. Modification of Bent

One gram of 3-aminopropyltriethoxysilane, the silane coupling agent, and 1.0 g of Bent were added into 50 mL of methylbenzene. The mixture solution was heated to 60 °C and stirred for 5 min. After being dried at 80 °C in a vacuum oven, the as-prepared solid was ground to a powder. Next, 1.0 g of the sample was added into 50 mL of deionized water, followed by adding 2 g of PAA and a certain amount of glutaraldehyde. At room temperature, the reaction was held steady for 5 h. Subsequently, the precipitate was sequentially filtered and rinsed with deionized water. Being dried for 6 h at 80 °C in a vacuum oven, the solid was grinded to pass through a 200 mesh sieve. The as-prepared product was represented as Bent-PAA.

### 2.3. Characterizations

XRD patterns were obtained using a BRUKER AXS D8 X-ray diffractometer (Karlsruhe, Germany). The X-ray generator was operated at 40 kV and 40 mA using CuK_α_ (λ = 1.540598 Å). Data were collected with a *ω* scan width of 0.02°. FTIR spectra were recorded on a PE Spectrum One spectrometer (Shelton, CT, USA). Thermal studies (TG/DTA) were conducted on Perkin Elmer (Shelton, CT, USA) in air at a heating rate of 10 °C min^−1^. SEM was collected using a Philips XL-30 FEG SEM instrument at 25 kV (Eindhoven, The Netherlands).

### 2.4. Batch Adsorption Experiments

The batch adsorption experiments were operated by a Dongpeng SHA-C water bath shaker (Jintan, China). The effect of experimental parameters, such as the initial concentration (*c*_0_) of AB10B, pH, experimental temperature (*T*), and contact time (*t*), on the adsorption amount was studied according to Table 1. Here, the adsorption amount of Bent-PAA for AB10B can be calculated by the following equation:

(1) $$q_{e}=\left(c_{0}-c_{e}\right)V/m,$$

where *c_e_* is the equilibrium concentration of AB10B in solution (mg L^−1^), *V* is the volume of solution (L), and *m* is the mass of the adsorbent (g). All experiments were performed in triplicate and the results are reported as the mean ± standard deviation.

## 3. Results and Discussion

### 3.1. Bent-PAA Characterization

**Figure 2:**
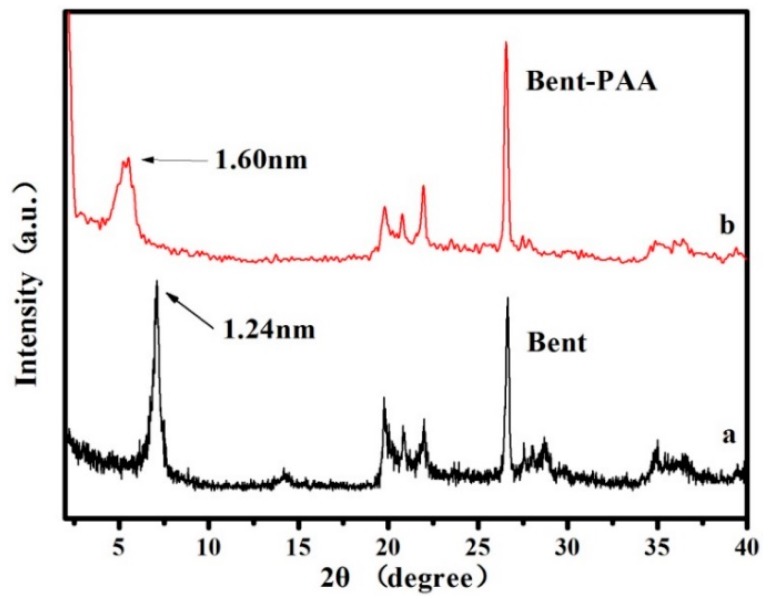
The XRD patterns of Bent-PAA (**a**) and Bent (**b**).

Figure 2 shows the XRD patterns of Bent and Bent-PAA. The distance between layers of bentonite can be determined based on the position of the characteristic reflection peak (001) in the XRD patterns. In Figure 2a, the XRD pattern of Bent had a characteristic (001) reflection at approximately 7.10° (2θ) [25], while the peak position shifted to 5.51° (2θ) after Bent was modified with PAA. Results calculated by the Bragg equation showed that the shift in the distance between layers was from 1.24 nm (Bent) to 1.60 nm (Bent-PAA) when Bent was modified with PAA. It can be inferred that the monolayer of PAA molecules was arranged parallel to the interlayer space of Bent.

**Figure 3:**
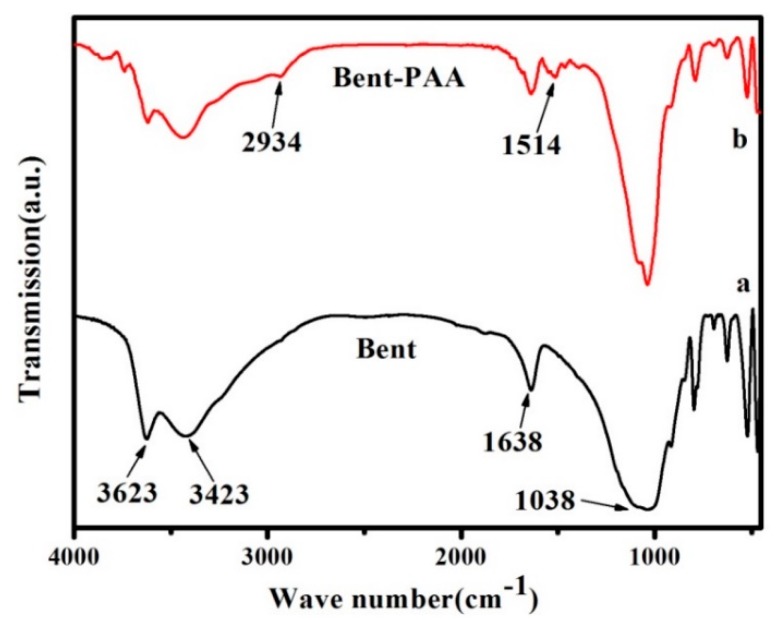
The FTIR spectra of Bent (**a**) and Bent-PAA (**b**).

Figure 3 shows the FTIR spectra of Bent and Bent-PAA. In Figure 3a, the bands at 3623 and 3423 cm^−1^ were attributed to the stretching vibration of structural hydroxyls (Si–OH, Al–OH) and the physical absorption of water (H–OH), respectively [26,27], with a corresponding deformation band (H–O–H) at 1638 cm^−1^ [28]. The characteristic bands at 1038, 798, 519, and 465 cm^−1^ were ascribed to Si–O stretching vibration from the Si–O–Si tetrahedron, (Al, Mg) –OH vibration mode, Si–O–Al deformation, and the angular deformation of Si–O–Si, respectively [29]. Spectra of Bent-PAA possessed an obvious peak at 1514 cm^−1^ caused by the bending vibrations of –NH_2_ while Bent did not. The peak of –CH_2_– asymmetric stretching at 2934 cm^−1^ also confirmed the successful modification of Bent with PAA.

**Figure 4:**
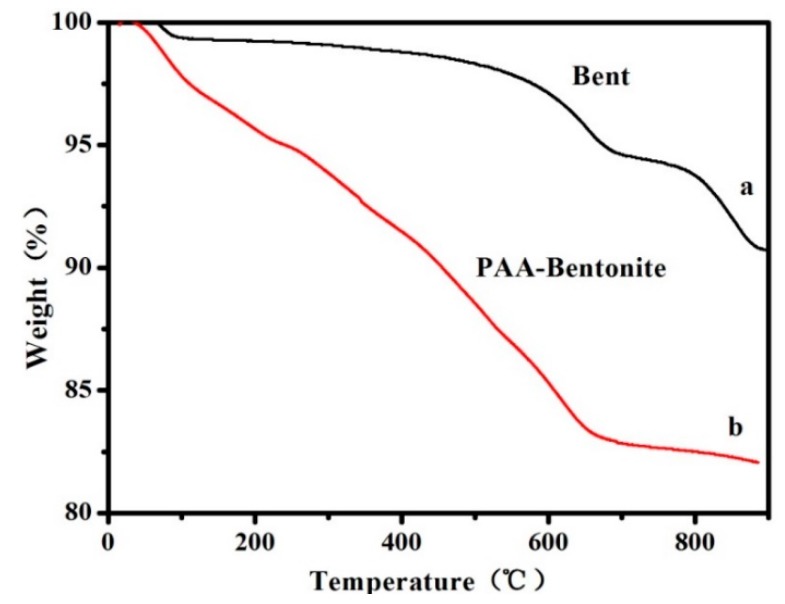
The TGA curves of Bent (**a**) and Bent-PAA (**b**).

The thermodynamic stability of Bent and Bent-PAA has been investigated by thermogravimetric analysis (TGA). In Figure 4a, a 6% weight loss of Bent appeared at 680 °C, caused by the dehydroxylation of the silicate layers, while the weight loss of Bent-PAA was much greater than that of Bent, attributed to the loss of PAA molecules modified on Bent. The results directly reflected the successful modification of PAA on Bent.

**Figure 5:**
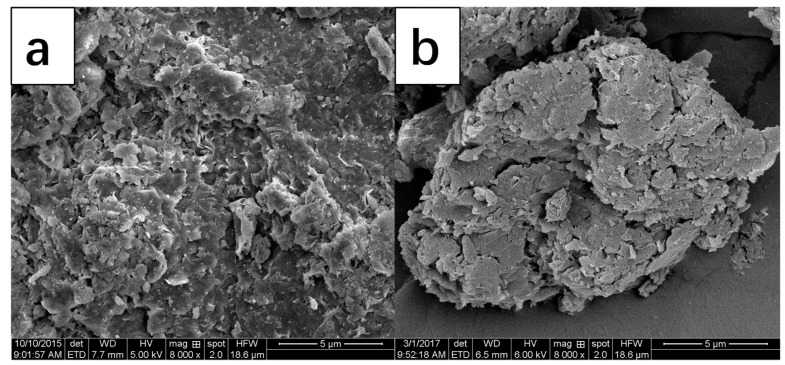
SEM images of Bent (**a**) and Bent-PAA (**b**).

In order to obtain information about the shape and size, SEM measurements of Bent and Bent-PAA have been done. Results are shown in Figure 5. Compared with the surface of Bent, that of Bent-PAA showed a much more regular, layered structure. Obviously, considerably larger cracks were formed.

### 3.2. Adsorption Results

#### 3.2.1. Effect of pH

**Figure 6:**
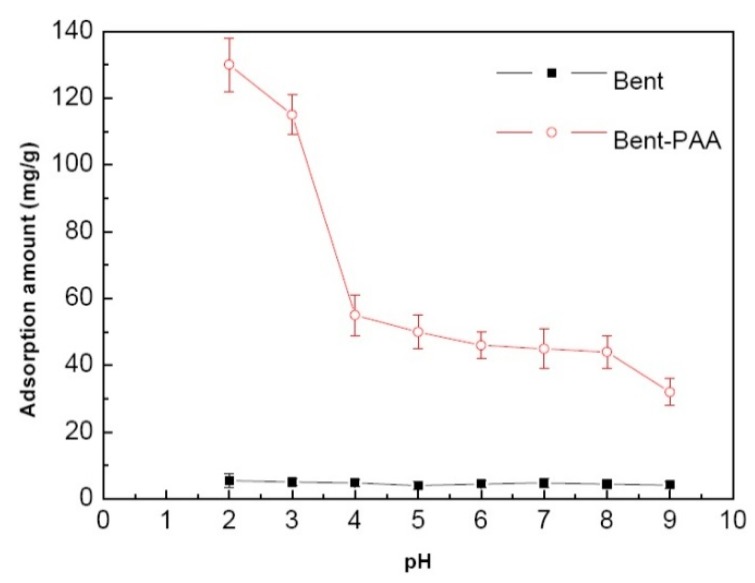
Effect of the solution pH on the adsorption amount of Bent and Bent-PAA for AB10B. Adsorption conditions: adsorbent dosage: 1.0 g L^−1^; at 298 K and pH 2–9; Contact time: 480 min; Dye concentration: 200 mg L^−1^.

The effect of solution pH from 2.0 to 9.0 on the adsorption amount of Bent and Bent-PAA has been investigated. Results are shown in Figure 6. The adsorption amount of Bent for AB10B was nearly zero within the whole designed pH range, indicating that Bent had no adsorption ability for AB10B. Compared with Bent, the adsorption amount of Bent-PAA for AB10B obviously increased and depended on the pH value. As for Bent-PAA, *q_e_* decreased quickly within the value of pH ranging from 2.0 to 4.0. After that, *q_e_* decreased gradually. This phenomenon may be caused by the effect of hydrogen ions on the positive sites of the adsorbent. At a lower pH, the more hydrogen ions in solution made the surface of Bent-PAA covered with more positive charges, which was beneficial for the electrostatic attraction between the positive charges of Bent-PAA and the negative charges of AB10B. When pH increased, the number of positive charges loaded on the surface of Bent-PAA reduced, which led to a decrease in the number of interaction sites of Bent-PAA with AB10B. Thus, the adsorption amount of Bent-PAA for AB10B decreased with the rise in pH. Results demonstrated that the graft of PAA contributed to the improvement of adsorption amount.

#### 3.2.2. Effect of Contact Time

**Figure 7:**
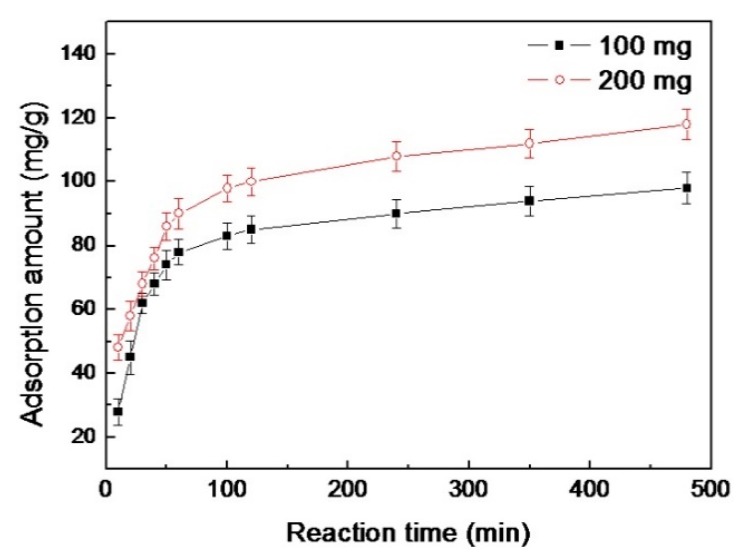
Effect of contact time of Bent-PAA with AB10B on the adsorption amount. Adsorption conditions: adsorbent dosage: 1.0 g L^−1^; at 298 K and pH 3; Contact time: 0–480 min; Dye concentration: 100 and 200 mg L^−1^.

The effect of contact time on the adsorption amount of Bent-PAA for AB10B has been investigated as shown in Figure 7. In the first 60 min, the adsorption amount increased quickly and gradually reached equilibrium. It seems clear that, during the first period, the bare surface was easier to cover with AB10B, which caused the obvious increase in the adsorption amount of AB10B. After that, the next AB10B molecules should choose the limited bare surface to be loaded, which led to a decrease in the adsorption rate.

#### 3.2.3. Effect of Initial Concentration of AB10B

**Figure 8:**
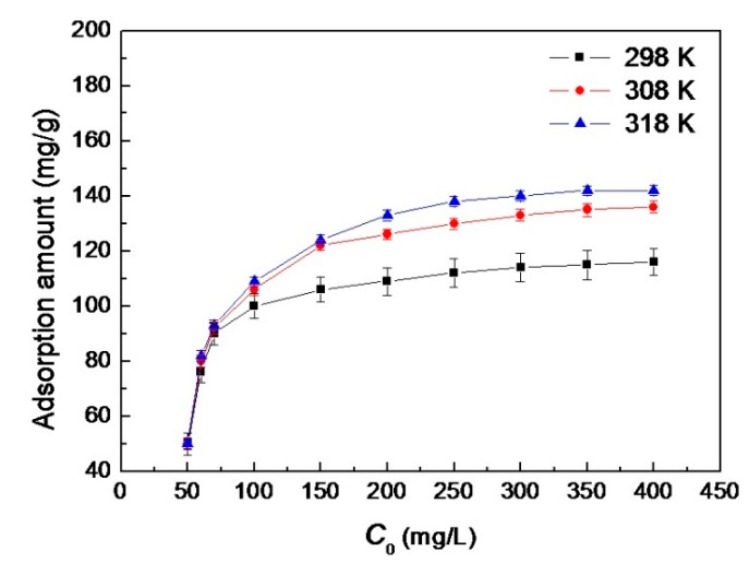
Effect of *c*_0_ of AB10B on the adsorption amount of Bent-PAA for AB10B at different temperatures. Adsorption conditions: adsorbent dosage: 1.0 g L^−1^; at 298, 308 and 318 K and pH 3; Contact time: 480 min; Dye concentration: 50–400 mg L^−1^.

The effect of initial concentration of AB10B on the adsorption amount of Bent-PAA has been investigated at different temperatures (25, 35, 45 °C). Results are shown in Figure 8. The adsorption amount of AB10B increased quickly within the initial period with the increase of initial concentration of AB10B and then gradually became more stable. When the concentration of AB10B was in a lower concentration range, the ratio of the absorption sites of Bent-PAA to AB10B was higher, leading to a higher removal rate of AB10B. When the concentration of AB10B was higher, the adsorption sites were gradually saturated, which led to the relatively lower adsorption rate of AB10B on Bent-PAA.

#### 3.2.4. Effect of Temperature

The effect of temperature on the adsorption amount of Bent-PAA for AB10B can also be seen in Figure 8. The adsorption amount of Bent-PAA for AB10B was higher at higher temperatures, which indicated that the adsorption interaction was an endothermic process. Moreover, the thermodynamic parameters for the adsorption processes have been calculated according to a previous method [19]. Here, ∆*G* was −23.94 kJ mol^−1^, ∆*H* was −14.22 kJ mol^−1^ and ∆*S* was 35.62 J mol^−1^ K^−1^. The negative values of ∆*G* and ∆*H* with the positive value of ∆*S* indicated that the adsorption process was spontaneous and exothermal, driven by the increase in entropy.

#### 3.2.5. Adsorption Isotherm

Here, the adsorption data have been fitted to a couple of widely used adsorption isotherms, namely, the Langmuir and Freundlich adsorption isotherm models. Langmuir adsorption isotherm can be expressed as follows:

(2) $$\frac{c_{e}}{q_{e}}=\frac{1}{K_{L}q_{m}}+\frac{c_{e}}{q_{m}},$$

where *c_e_* is the equilibrium concentration of adsorbate (mg/L), *q_e_* is the adsorption amount of adsorbent (mg/g), *q_m_* is the maximum adsorption amount in theory, and K*_L_* is the Langmuir constant related to the affinity of binding sites.

However, the Freundlich model is an empirical formula suitable for heterogeneous surfaces. The corresponding equation is given below:

(3) $$\ln q_{e}=\ln K_{F}+1/n\ln c_{e},$$

where K*_F_* and n are Freundlich constants. K*_F_* indicates the relative adsorption capability and n is related to adsorption intensity.

**Figure 9:**
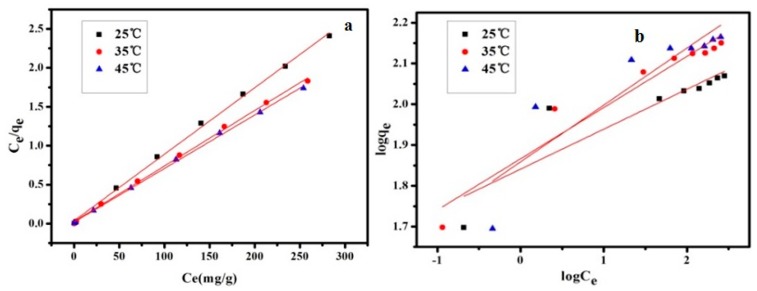
Isotherms of Bent-PAA for AB10B according to Langmuir model (**a**) and Freundlich model (**b**). Adsorption conditions: adsorbent dosage: 1.0 g L^−1^; at 298, 308 and 318 K and pH 3; Contact time: 480 min; Dye concentration: 50–400 mg L^−1^.

The adsorption data were fitted to Equations (2) and (3) and the linear simulations were shown in Figure 9. In addition, corresponding isotherm parameters for the two models are summarized in Table 2. The correlation of the Langmuir model was stronger than that of the Freundlich model based on the regression coefficients (*R*^2^), suggesting a monolayer adsorption. What is more, the theoretical *q_m_* was in agreement with the experimental *q_m_* as shown in Figure 7.

#### 3.2.6. Adsorption Kinetics

The mechanism of adsorption about the real adsorption process was investigated by pseudo-first-order and pseudo-second-order kinetic models, respectively.

The pseudo-first-order kinetic model is expressed as the following two expressions:

(4) $$dq_{t}/dt=k_{1}\left(q_{e}-q_{t}\right),$$

(5) $$\ln\left(q_{e}-q_{t}\right)=\ln q_{e}-k_{1}t,$$

where *q_e_*, adsorption amount of the adsorbents for AB10B at equilibrium time; *q_t_* adsorption amount of the adsorbents for AB10B at any time; k_1_, the equilibrium rate constant of pseudo-first-order kinetic model.

The pseudo-second order process can be written as follows:

(6) $$\frac{t}{q_{t}}=\frac{1}{k_{2}q_{e}2}+\frac{t}{q_{e}},$$

where k_2_ is the equilibrium rate constant of the pseudo-second-order model.

**Figure 10:**
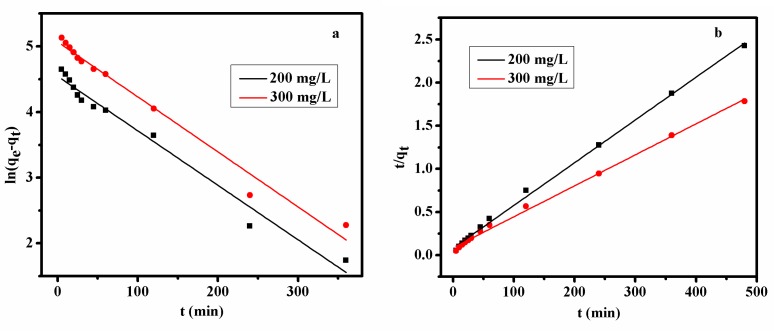
The linear fitted data according to pseudo-first order model (**a**) and pseudo-second order (**b**) Adsorption conditions: adsorbent dosage: 1.0 g L^−1^; at 298, 308 and 318 K and pH 2; Contact time: 480 min; Dye concentration: 200 and 300 mg L^−1^.

The fitted results are shown in Figure 10 and the related parameters obtained from the fitting curves are listed in Table 3. In the case of the pseudo-first-order kinetic model, *R*^2^ was lower than 0.98, while *R*^2^ was over 0.99 in the pseudo-second-order kinetic model. Compared with the pseudo-first-order kinetic model, the pseudo-second-order kinetic model was more suitable for describing the adsorption process.

#### 3.2.7. Regeneration and Reusability

The as-prepared adsorbent after saturated adsorption could be easily regenerated by using dilute NaOH aqueous solutions. The adsorption capacity for the reused adsorbent could remain 95%.

## 4. Conclusions

Bent was functionalized with polyene propyl amine. The characterization results of XRD, FTIR, TGA, and SEM demonstrated that PAA was modified successfully onto interlayers of bentonite. Then the adsorption capability of Bent-PAA was tested against the removal of AB10B from water. Compared with Bent, the adsorption amount of Bent-PAA was excellent. The adsorption isotherm was also studied and the equilibrium data could be well described by the Langmuir adsorption isotherm model. Thus, it is expected that Bent-PAA will be an ideal adsorbent for the fast removal of AB10B from wastewater.

## Figures and Tables

**Table 1:** Batch sorption experiments parameters.

Adsorbent	*t* (min)	pH	*c*_0_ (mg L^−1^)	*m*_a_ (g)	*V*_s_ (mL)	*T* (°C)	*S* (rpm)
Bent-PAA	480	2–9	200	0.03	30	25	200
	10–480	2	100–200	0.03	30	25	200
	480	2	50–400	0.03	30	25–45	200

*m*_a_, *V*_s_, *T*, and *S* represent the mass of adsorbent, the volume of solutions, experimental temperature, and rotational speed, respectively.

**Table 2:** Adsorption parameters of Bent-PAA for AB10B according to Langmuir model and Freundlich model.

*T* (°C)	*q*_exp_ (mg/g)	Langmuir			Freundlich		
		*R* ^2^	*q_m_* (mg/g)	*K*_L_ (L/mg)	*R* ^2^	*K*_F_ (L/g)	*n*
25	117.28	0.998	128	0.222	0.782	6.3	10.21
35	141.6	0.998	142.86	0.269	0.929	6.46	7.97
45	144.08	0.999	166.67	0.3	0.788	6.41	7.17

**Table 3:** The fitted parameters for the adsorption of Bent-PAA for AB10B.

	Pseudo-First-Order Model		Pseudo-Second-Order Model	
C (mg/L)	*R* ^2^	k_1_ (mg^−1^)	*R* ^2^	k_2_ (g·mg^−1^·min^−1^)
200	0.976	0.0083	0.998	0.00495
300	0.979	0.0084	0.997	0.00359
